# Biological Insights into Myeloma and Other B Cell Malignancies

**DOI:** 10.1155/2016/5218093

**Published:** 2016-02-16

**Authors:** Mariateresa Fulciniti, Nicola Amodio, Michele Cea, Patricia Maiso, Abdel Kareem Azab

**Affiliations:** ^1^Dana-Farber Cancer Institute, Harvard Medical School, Boston, MA 02215, USA; ^2^Department of Experimental and Clinical Medicine, Magna Graecia University of Catanzaro, Catanzaro, Italy; ^3^University of Genoa, Genoa, Italy; ^4^University of Navarra, Pamplona, Spain; ^5^Washington University, Saint Louis, MO 63108, USA

Multiple myeloma (MM) is a hematologic cancer characterized by the accumulation of malignant plasma cells in the bone marrow, which causes bone destruction and marrow failure [1]. Despite the fact that clinical introduction of novel drugs has led to a dramatic improvement of disease, MM ultimately relapses and remains an incurable disease [2]. Consistently, research in this field has dramatically grown, as demonstrated by the increased rate of publications during the last decade. Original research and review articles published in this special issue focus on identification of new targets and pathways for prognostic and therapeutic application in myeloma, as well as validation of novel antitumor agents.

*(1) Diagnosis, Monitoring, and Prognosis of MM*. Extensive gene expression profile analysis has provided interesting insight into the disease biology, and its correlation with clinical outcome has opened a new direction for risk stratification as well as novel target and therapy identification. Prognostic models such as the Durie-Salmon staging system and International Staging System (ISS) are available and account for the disease burden [3]; however, the original analysis of ISS system did not include Chinese patients' data and take into account the recent introduction of novel agents. The data provided in the manuscript by J. Lu et al. represent the first multicenter retrospective study analyzing ISS value in a large number of unselected Chinese myeloma patients. The results demonstrate that ISS still has prognostic value in Chinese patients with MM, but not in patients receiving bortezomib-based therapy. Therefore, further studies are needed to develop more suitable and robust stratification systems to predict prognosis and optimize treatment strategy early during the course of the disease.

Minimal residual disease (MRD) has emerged as one of the most relevant prognostic factors in MM and should be included in a new definition of complete response (CR) [4]. In the manuscript by M. Fulciniti et al., the authors reviewed current definition of deep response in MM, advantages and limitations of current MRD assessment assays, and clinical evidences for MRD monitoring as a prognostic tool for therapeutic decisions in MM, providing the rationale for the use of MRD assessment in the evolving MM clinical paradigm. 

*(2) Role of the Bone Marrow Milieu*. The bone marrow (BM) microenvironment plays a crucial role in MM pathogenesis. MM cells reside in and dynamically interact with various subsets of cells in the BM including mesenchymal cells (MSCs), osteoclasts (OCLs), osteoblasts (OBLs), and vascular endothelial cells, which support growth and survival of the tumor cells and lead to development of drug resistance [5]. These cells not only physically interact with MM cells but also secrete growth and/or antiapoptotic factors. The cross talk between MM cells with bone marrow stromal cells or other cellular components is finely tuned by a plethora of cytokines, growth-factor, and other molecules which can be released either by MM cells or by cells of the BM microenvironment. Several studies have demonstrated deregulation of the cellular and humoral components of the BM in MM including increased OCL activity and inhibited OBL activity, and both are involved in the pathophysiology of the bone lesions in MM [6].

The review by F. Accardi et al. summarizes the preclinical and clinical evidence on the effects of bortezomib and other new Proteasome Inhibitors (PIs) on myeloma bone disease. Osteoclastic formation and activity are inhibited by PIs, mainly through the blockade of RANKL signaling pathway in the osteoclast progenitors. However, the more significant impact of the bone remodeling by this class of drugs is the capacity to stimulate either the osteogenic differentiation of MSC or the osteoblastic function, leading to the consequent bone formation with a considerable anabolic effect. Osteocytes are also possible targets of PIs with a stimulatory effect on their viability. The preclinical evidence, thus, is confirmed in MM patients treated with bortezomib and more recently with carfilzomib. An improvement of the bone remodeling markers was observed in the patients treated with PIs. The histomorphometric data in MM patients treated with bortezomib prominently indicated that PIs can stimulate the bone formation process and induce the bone regeneration process. Bone healing, as well as an increase in the BMD, has also been reported in some of the patients treated with bortezomib. Overall, the literature data support the use of these drugs to restore bone integrity in MM patients.

MM cells are cradled within the BM microenvironment by an array of adhesive interactions between the BM extracellular matrix (ECM) components and a variety of adhesion molecules on the surface of MM cells, which results in the “cell adhesion-mediated drug resistance” (CAM-DR) thought to be one of the major mechanisms by which MM cells escape the cytotoxic effects of therapeutic agents [7]. MM is characterized by continuous spread of cancer cells at different sites of the bone marrow, through continuous cell trafficking including adhesion of myeloma cells to vascular wall. This process requires the presence of P-selectin on the endothelium and stroma, as well as PSGL-1 on tumor cells. PSGL-1 was previously suggested as a novel target for immunotherapy in MM using small molecule inhibitor which demonstrated sensitization of MM cells to therapy, controlling tumor growth and dissemination. However, poor pharmacokinetic profile with a very short half-life may hold up further usage of this drug in MM. The study by B. Muz et al. demonstrates that inhibition of P-selectin/PSGL-1 axis using humanized monoclonal antibodies is a promising approach for the treatment of MM with high efficacy in inhibition of P-selectin/PSGL-1 interactions and sensitization of MM cells to therapy, along with favorable pharmacokinetics.

Angiogenesis is fundamental to tumor growth and spread in many hematological disorders, particularly MM [8]. The angiogenic potential of MM is regulated by several pro- and antiangiogenesis cytokines produced by myeloma cells and other cell types in the tumor microenvironment. The study by T. Valković et al. determines the plasma levels of monocyte chemotactic protein-1 (MCP-1), as well as its possible association with angiogenesis, in 45 newly diagnosed MM patients and 24 healthy controls. The manuscript reveals a positive association between plasma MCP-1 levels, angiogenesis, and clinical features in patients with MM. However, additional prospective studies with a respectable number of patients should be performed to authenticate these results and establish MCP-1 as a possible target of active treatment.

*(3) Genetic and Epigenetic Abnormalities*. MM cells accumulate various genetic and epigenetic abnormalities that drive the malignant phenotype and confer distinct biologic sequelae and disease outcomes [9].

miRNA are important transcriptome modifiers that play important role in myeloma tumor progression, survival, and development of drug resistance [10]. In their review, Raimondi et al. discuss the role of endogenous noncoding RNAs as microRNAs as a novel class of regulators of the intercellular communication between MM cells and other cells of the BM milieu, focusing on the therapeutic potential of experimental strategies aimed at modulating microRNA levels in MM cells and capable of overcoming the tumor-promoting BM microenvironment.

Moreover, S.-F. Lin and W.-C. Yang review molecular mechanisms underlying MM development, progression, and resistance to treatment, further highlighting the relevance of microRNAs as well as immune dysfunctions and conventional or novel therapies targeting such vulnerabilities. Overall, authors advocate that understanding the genomic landscape of MM deserves more attention in the prospect of developing personalized and effective targeted therapies that overcome resistance to currently used anti-MM drugs.

In addition to microRNAs, dysregulation of transcription factors (TFs) features prominently in the biology of MM and B cell malignancies. TFs are the downstream effectors of signaling pathways within cells, which receive growth and other signals from the microenvironment; they also regulate cellular homeostasis as well as cell survival and proliferation. On this basis, mutations in TF genes or dysregulation of TFs' expression could play a significant role in cancer pathogenesis and drug resistance. The TF EBF1 is the master regulator of the specification, development, and maintenance of the B-lymphoid lineage, and perturbations of EBF1 expression and/or functions have been associated with the development of B cell malignancies [11]. In this regard, M. Mesuraca et al. have reviewed the role of two zinc finger proteins, namely, ZNF423 and ZNF521, as potent inhibitors of EBF1 and as likely contributing to the development of B cell leukaemia diseases.

Genome instability, defined by higher rate of genomic changes acquisition per cell division compared to normal cells, represents a prominent feature of MM cells [12]. M. Cea et al. provide a comprehensive overview of the current knowledge of genomic instability in MM both in terms of its contribution to disease development and progression and in terms of possible relevance as therapeutic target. The paper describes mechanisms by which genetic aberrations give rise to multiple pathogenic events required for myelomagenesis and concludes with a discussion of the clinical applications of these findings in MM patients.

Altogether, the data presented in this special issue may contribute to increasing our current knowledge of the biology of MM and B cell malignancies.

*Mariateresa Fulciniti* *Nicola Amodio* *Michele Cea* *Patricia Maiso* *Abdel Kareem Azab*
